# Interactions Between Rhythmic and Feature Predictions to Create Parallel Time-Content Associations

**DOI:** 10.3389/fnins.2019.00791

**Published:** 2019-08-02

**Authors:** Sanne ten Oever, Alexander T. Sack

**Affiliations:** ^1^Department of Cognitive Neuroscience, Faculty of Psychology and Neuroscience, Maastricht University, Maastricht, Netherlands; ^2^Maastricht Brain Imaging Centre, Maastricht, Netherlands

**Keywords:** prediction, EEG, temporal information, rhythm, adaptation

## Abstract

The brain is inherently proactive, constantly predicting the when (moment) and what (content) of future input in order to optimize information processing. Previous research on such predictions has mainly studied the “when” or “what” domain separately, missing to investigate the potential integration of both types of predictive information. In the absence of such integration, temporal cues are assumed to enhance any upcoming content at the predicted moment in time (general temporal predictor). However, if the when and what prediction domain were integrated, a much more flexible neural mechanism may be proposed in which temporal-feature interactions would allow for the creation of multiple concurrent time-content predictions (parallel time-content predictor). Here, we used a temporal association paradigm in two experiments in which sound identity was systematically paired with a specific time delay after the offset of a rhythmic visual input stream. In Experiment 1, we revealed that participants associated the time delay of presentation with the identity of the sound. In Experiment 2, we unexpectedly found that the strength of this temporal association was *negatively* related to the EEG steady-state evoked responses (SSVEP) in preceding trials, showing that after high neuronal responses participants responded inconsistent with the time-content associations, similar to adaptation mechanisms. In this experiment, time-content associations were only present for low SSVEP responses in previous trials. These results tentatively show that it is possible to represent multiple time-content paired predictions in parallel, however, future research is needed to investigate this interaction further.

## Introduction

Rhythmic stimulus input provides predictable temporal structure on the basis of which the state of the brain can be adapted in order to optimize processing of upcoming stimuli. It has been proposed that attention can be directed to the isochronous moments within such a rhythmic input stream at which sensory input can be expected (Jones, 1976; Jones et al., 2002; Schroeder and Lakatos, 2009; Nobre and van Ede, 2018). In light of these theories, the majority of studies investigating the role of rhythmic temporal information have intuitively focused on the use of rhythm to inform about the when, that is, the most likely arrival time of (any) sensory information (Nobre et al., 2007; Rohenkohl et al., 2012; Lawrance et al., 2014). Typically, the sensory processing of information at an expected time point and an unexpected time point are contrasted. Indeed, it has been shown that participants are better at detecting a subthreshold sound when it is preceded by rhythmic input (Ten Oever et al., 2014) and that sound discrimination performance improves when the sound’s arrival time follows the timing of the rhythmic stream (Jones et al., 2002). However, in a natural environment many events occur in parallel and different arrival times might signal different predicted content. It is still unknown whether unified parallel expectations are created interactively using both predictions from rhythmic temporal information (when) and predictions about specific content features (what).

Within the general temporal predictor framework, any stimulus occurring at the exact time point when relevant information is expected will be processed most optimally. Alternatively, in the parallel time-content prediction framework, temporal information interacts with feature information to allow for a more flexible prediction mechanism. In the latter, behavior will only be optimized when a certain content matches the prediction in the temporal as well as the feature prediction domain. In such a scenario, it would be possible to have different time points associated with different contents in parallel. The studies investigating the interaction between feature (or space) and temporal predictions have shown that temporal information is mostly beneficial in cases where a content prediction is available (Zaehle et al., 2009; Rimmele et al., 2011; Morillon et al., 2016). Additionally, in memory studies, supra-second cue-target delays have been shown to be used to recall content-specific information (Molet and Miller, 2014; van Ede et al., 2016; Cravo et al., 2017; van de Ven et al., 2017). These studies suggest that we should not think of temporal information as merely enhancing any sensory information arriving at a specific moment in time. Instead, time likely acts as another cue for inferring the content of upcoming information. However, the described studies have either not used parallel predictions (merely absence or presence of a prediction) or used paradigms with supra-second cue-target delays which have been shown to act on different neuronal mechanisms compared to rhythms (Rohenkohl et al., 2011; Breska and Deouell, 2014). In sum, the existence of parallel time-content predictions during rhythmic stimulus input is still elusive.

Rhythmic input has been shown to modulate neuronal responses such that oscillations align to the rhythmic input stream (Lakatos et al., 2005). The behavioral consequences of such entrainment are that stimuli presented within the rhythmic structure fall on an excitable point of an oscillation, thereby boosting information processing, and behavioral performance (Canolty et al., 2006; Schroeder and Lakatos, 2009; Haegens et al., 2011; Haegens and Golumbic, 2017). Based on this neuronal mechanism, it is predicted that in-phase stimuli should be processed better than out-of-phase stimuli. In contrast, high gamma power as well as spiking activity related to the representation of different items have also been shown to cluster at different phases of an oscillation (O’Keefe and Recce, 1993; Lee et al., 2005; Bahramisharif et al., 2018). This suggest that depending on the phase of the input different neuronal populations are active (Lisman and Jensen, 2013), biasing the overall percept to one or the other item (Ten Oever and Sack, 2015). Such activity clustering as well as a phase dependent perceptual bias has been reported for phases as much as half a cycle apart (Ten Oever and Sack, 2015; Bahramisharif et al., 2018). In the current study, we aimed to investigate if this perceptual bias can be induced by systematically presenting different items at time points half a cycle apart after an entrainment train. As such, we investigate whether it is possible that within one cycle of a rhythmic input stream different time points/phases are associated with different content items, resulting in a systematic response bias toward one or the other item at a given phase. This bias can then be compared with the overall discrimination performance for stimuli at the expected versus unexpected time of the entrainment stream. Investigating both the possibility of time-content associations as well as the relation between temporal expectancy and accuracy during a discrimination task can contribute to understanding the neuronal mechanisms for coding statistical regularities.

Here, we present two temporal association experiments. In both experiments specific sound categories were systematically presented at different time delays after presenting a rhythmic visual input stream in order to investigate if temporal information is used as a cue for content. If the brain integrates the predictions about the temporal and about the acoustic features, parallel time/sound-identity predictions should be formed, and subsequently, performance should be better when sound category A is presented at its associated time point A as compared to time point B (and vice versa for sound category B).

## Materials and Methods

### Participants

Thirty and 40 participants completed Experiments 1 and 2, respectively, (Experiment 1: mean age: 20, range 18–26, 5 males. Experiment 2: mean age: 26, range: 18–56, 13 males). All reported to have normal or corrected-to-normal vision and unimpaired hearing. In Experiment 2, three participants were excluded. For two participants the EEG data quality was too low. One participant did not finish the full session. All participants were informed about the study and gave written informed consent. The experiment was approved by the local ethical committee at Faculty of Psychology and Neuroscience Maastricht University (ethical approval number: ECP-127 14_04_2013). Participants were compensated for their time by either vouchers or participation credits.

### Experiment 1

#### Stimuli and Procedure

**FIGURE 1:**
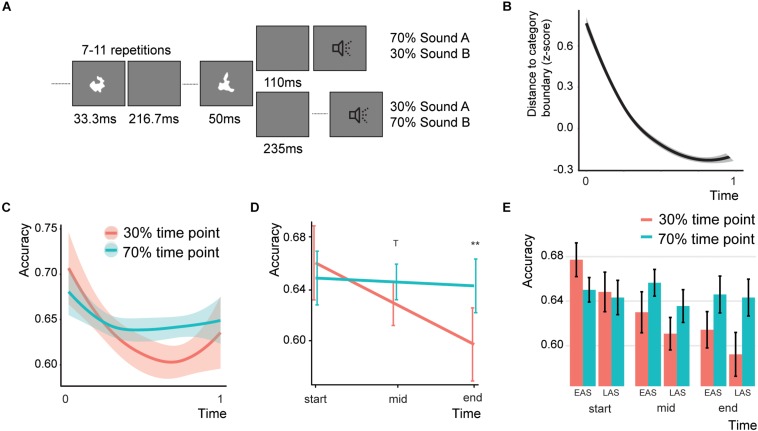
Design and behavioral results of Experiment 1. **(A)** Typical trial for Experiment 1. **(B)** The difficulty over the course of the trial. The higher the distance, the more difficult the task. **(C)** Results of Experiment 1 over the course of the experiment (smoothed with two-span loess smoothing in R). Shaded areas represent standard errors of the mean. **(D)** Predicted effects for the Association^*^Time interaction. Error bars indicate the 95% confidence intervals. T indicates a trend (*p* < 0.1) and double asterisks indicate significance (*p* < 0.01). **(E)** Bar graph for the Association effect, showing the data for both sound types (EAS = early association sound and LAS = late association sound) and three equally space time bins. Error bars represent the standard error of the mean. Note that the accuracy for the 70% time point does not change as the difficulty level is adjusted based on the performance on the 70% association trials.

A sequence of random visual stimuli were presented in a 4 Hz rhythmic fashion (presented for 33.34 ms; 192 pixels wide; Linden et al., 2003). The amount of visual stimuli was varied between 7 and 11 stimuli at an approximate hazard rate (due to rounding; 7: 63.3%; 8: 23.3%; 9: 8.6%; 10: 3.2%; 11:1.2%; Figure 1A). The last visual stimulus was always the same (and lasted 50 ms), and was followed by an auditory stimulus. We used ripple sounds as auditory stimuli (see e.g., Kowalski et al., 1996). These sounds resemble the spectral and temporal properties of natural sounds including speech. The sounds consisted of 50 logarithmically spaced sinusoids spanning 5 octaves that varied in spectral and temporal modulations. They varied in amplitude (100% modulation) and velocity (3, 4, or 5 cycles/s). We fixed the density of the sounds at 0.25 cycles/octave, indicating the frequency modulation. The fundamental frequency of the sound determined whether the sound was part of an arbitrary distinction between a “category A” and a “category B” sound. We fixed this category boundary at 500 Hz. We created a total of 201 sounds, 100 at either side of the boundary with fixed intervals in the mel scale (ranging between 182 and 932 Hz, corresponding to 260 and 954 mel). Sounds were either presented at 110 ms or at 235 ms after the onset of the last visual stimulus (stimulus onset asynchrony of 160 and 285 ms, respectively). We specifically choose the two time points to be at half a cycle distance to maximize the phase difference between the two time points.

Participants were required to identify the category of the sounds (either A or B). To maintain task difficulty, we implemented a staircase procedure by which the sound categorization became more and more difficult. Initially, the value of the difficulty level was 60 away from the category boundary (thus higher numbers are easier). We employed a 1-up-1-down staircase. Thus, at each mistake or correct answer the categorization was made easier or more difficult, respectively. However, only information of the associated time point was used for the staircase adaptation (see below). The stepsize of the difficulty change was initially 10, but changed to 5 and 1 after 6 and 10 switches, respectively. In this way participants were exposed to a constant difficulty level by which we intended to optimize the association learning. As expected, the difficulty level increased over the course of the trials (Figure 1B).

To create an association between time point and sound category we presented 70% of all category A sounds after 110 ms and 30% at 235 ms after the last visual stimulus offset. For category B sounds 70% and 30% of the sounds were presented at 235 and 110 ms, respectively. To enhance the association learning we only gave feedback to the participants when the sound was presented at the associated time point. This feedback consisted of the fixation cross turning either red or green (144 ms). The fixation cross was on for the whole experiment (but with a white color). While the temporal delays that we used here can typically be detected above chance in a forced-choice temporal order experiment (Vroomen and Keetels, 2010), none of the participants reported being aware of the temporal offsets after explicitly asking them about the design of the experiment. The next trial started between 1 and 1.5 s after the response. In total there were 6 blocks of 100 trials each. For half of our participants we reversed the association (category A: 325 ms; category B: 110 ms). For the analysis, we recoded the data for these participants. In the remainder of the experiment we will refer to the sounds presented at 70% at an inter-stimulus interval (ISI) of 110 ms as the early association sounds, and the sounds presented at 70% at an ISI of 235 ms as the late association sounds.

#### Analysis

Our objective was to investigate if over the course of the experiment participants started responding in correspondence with the time-content association. In order to investigate the development throughout the experiment, we performed the analysis on a single trial level. Specifically, we performed a generalized linear mixed model (glmer) with a binomial logit link function [using lme4 (Bates et al., 2014) under (R Core Team, 2013); a binomial logit function was required to account for the nominal accuracy data] including all trials using a random intercept for participants. Initially the following fixed factors were considered: association (non-associated (30%) and associated (70%) time point), Time (linear factor ranging between 0 for the first trial and 1 for the last trial), Sound Type (early association sounds and late association sound), and control variable Categorization difficulty (*z*-score of stepsize away from category boundary). Non-significant interaction effects were iteratively removed. On the final model we performed follow up tests with adjusted Bonferroni correction. Outliers – defined as the mean accuracy within or across a condition above the median ± 1.5 times the inter-quartile range – were removed. Effect sizes are reported as odds ratios (always of *z*-scored data or for binominal variables). If participants over the course of the experiment followed the association, we expect a significant Time^*^Association interaction (at the end, but not at the beginning of the experiment a higher accuracy for 70% associated time points). If instead participants were generally better for a specific time point (early or late), an interaction between sound type and association should be expected (if participants are better for the later time points: early association sounds have a higher accuracy for the 30% compared to the 70% non-associated time point and late association sounds have a higher accuracy for the 70% compared to the 30% associated time point).

### Experiment 2

#### Stimuli and Procedure

The procedure of Experiment 2 was identical to Experiment 1 with the following exceptions: (1) In Experiment 2 the step size of the sounds was decreased, creating a total of 401 sounds. (2) The ISI was increased to vary between 1.25 and 2 s to remove temporal structure in the task that could influence baseline EEG responses. (3) The first block in Experiment 2 was used to find the presentation rate at which the EEG response was highest. Originally, we hypothesized that by choosing a presentation rate with strong neuronal responses the temporal association effect would increase as the temporal structure can be estimated better. However, as evident in the results, this was not apparent. As such, in the first block we used presentation rates of 3.0, 3.5, 4.0, 4.6, 5.0, 5.5, 6.0, 6.7, 7.5, and 8.6 Hz for 10 trials each. The delay for the sounds was 110 ms after the offset of the sound for the early time point. For the late time point this was 110 ms + half the cycle of the used presentation rate (e.g., for 5.0 Hz this would be 110 + 0.5^*^200 = 210 ms). After the first block the individual presentation rate was determined (see section “Presentation Rate Determination”) and kept constant for the remainder of the experiment.

#### EEG Acquisition

Thirty two channel EEG data was recorded with a sampling rate of 2500 Hz with hardware online notch filters of 0.01–1000 Hz with Brainvision Recorder software (Brain Products), and BrainAmp MR Plus amplifier using the standard BrainCap MR. The following electrodes were used: Fp1, Fp2, F3, F4, C3, C4, P4, P5, O1, O2, F7, F8, T7, T8, P7, P8, Fz, Cz, Pz, Oz, FC1, FC2, Cp1, Cp2, FC5, FC6, CP5, Cp6, TP9, TP10, and POz. A vertical EOG channel was placed under the left eye, the ground was placed on AFz and the reference was Cz. Impedance for the reference and ground electrode was kept below 10 kΩ, for the other electrodes this was below 15 kΩ.

#### Presentation Rate Determination

All data was analyzed in Matlab version 2017a (Mathworks), using a combination of Fieldtrip (Oostenveld et al., 2011), EEGlab (Delorme and Makeig, 2004), and custom scripts. After the first block we estimated the presentation rate used for the rest of the experiment. As such, the data of the first block was notch filtered and epoched ranging from the presentation rate/5 (i.e., 5 cycles of data or 5 visual stimuli at the presentation rate) until sound onset and re-referenced to the average of all channels. This range was chosen to ensure that we captured time intervals where at least two visual stimuli were presented in the stream (minimal amount of visual stimuli was 7). Then a FFT with hanning taper was used to extract the complex Fourier spectra for frequencies ranging from 1 to 25 Hz. Subsequently both power and intertrial-coherence (ITC) were calculated for this range of frequencies for all presentation rates separately. The presentation rate with the highest ITC (averaged over channels Cz, FC1, FC2, Cp1, and Cp2) at the presentation rate was used for the remainder of the experiment. Central electrodes were chosen as the task at hand was an auditory task. However, it is unlikely that the EEG signal is purely related to the auditory generators but is likely also influenced by visual components. Further constrains were that the power also showed a peak at the presentation rate.

#### EEG Preprocessing

Data was epoched from −6 to +3 s around sound onset, demeaned and re-referenced to the average of all channels. Data was resampled to 256 Hz and trials with strong noise were removed via visual inspection. Eye blinks and muscle artifacts were removed using ICA. No qualitative differences were found additionally removing all trials with blinks in the time period of the last five visual stimuli before sound onset.

#### Behavioral Analysis

The behavioral analysis was identical as for Experiment 1. The block during the presentation rate determination was not included.

#### Subsequent Analysis

As the initial analysis did not result in a significant effect, we aimed to discover how the main change between Experiments 1 and 2 (the individualization in the presentation rate) could have explained the lack of time-content associations in Experiment 2. These analyses were *post hoc*, and statistically corrected as such, but their results should be interpreted taking this into account. We aimed to present these results in parallel with Experiment 1 to display the specificity of the effect, thereby promoting scientific transparency. We considered the following control variables that could modulate the effect: presentation rate, pure temporal predictions, adaptation effects.

#### Presentation Rate

To investigate the relation between the used presentation rate and the strength of the temporal association we repeated the final behavioral model of Experiment 1 (main Factors Association, Sound Type, Time, control factor Categorization difficulty, interaction of Association and Time), but adding the factor Presentation Rate and the interaction with all main and interaction effects. If not specified otherwise, for the following analyses the starting point is the final model of Experiment 1. The rational of this analysis was that for lower presentation rates, the difference between the early and late time point is stronger, potentially increasing the temporal association effect. In a second analysis we added the factor Presentation Rate change to the model. This factor entailed the absolute difference of the presentation rate and 4 Hz and the temporal association effect. The rational of the latter analysis was that it could be that the 4 Hz chosen in Experiment 1 may simply be the presentation rate with the best potential entrainment effect.

#### Pure Temporal Predictions

It could be expected that stimuli presented in-phase with the rhythmic visual stream would be processed better than rhythmic stimuli presented out-of-phase with this stream (Schroeder and Lakatos, 2009; Haegens and Golumbic, 2017). However, the varying presentation rates changed the absolute phase of the sound-onset times. Therefore, we calculated how far away the stimulus presentation time was from the most excitable phase point (0 = at the excitable phase point, pi = half a cycle away in either direction). Instead of using association, we recoded the factor association to Time Point (early time point and late time point) as it was more intuitive for the question asked. We used the same GLM as described for Experiment 1 using the fixed factors Sound Type, Phase Distance, Time Point, and Difficulty. We added the interaction of Phase Distance^*^Time Point. If it matters whether the sound was presented in-phase with the rhythm, participants with a low phase distance should be better at the early compared to the late time point, and vice versa for participants with a high phase distance. This should result in a Phase Distance^*^Time Point interaction.

#### Adaptation Effects – Within Subject

One other factor that could influence behavioral responses is adaptation. It has been shown that when repeatedly presenting a stimulus the response of participants’ is driven away from that stimulus category (Kanai and Verstraten, 2005; Daelli et al., 2010). For example, after adapting to a leftward motion, a rightward motion is perceived in ambiguous motion displays. This effect is caused by a desensitization of the neuronal populations representing that stimulus (Nelson, 1991; Sobotka and Ringo, 1994; Larsson and Smith, 2011). This desensitization is strongest when neuronal responses to the original stimuli were strongest (Li et al., 1993; Sobotka and Ringo, 1994). Based on adaptation effects it is predicted for the current experiment that stronger responses to the visual stimuli on the preceding trial could deter the behavioral responses from the association, consequently being more accurate for 30% compared to the 70% association trials. To investigate such effects, we extracted the steady-state visual evoked responses (SSVEP), by extracting for each trial the power at the frequency of the individual presentation rate. The data for each frequency estimated was epoched separately at 4 cycles until sound onset and subjected to a FFT with hanning tapers. The logarithm of the power was extracted for each trial individually for the frequency corresponding to each participants’ individual presentation rate. For four conditions the average SSVEP for the preceding seven trials was estimated: 30% incorrect trials, 30% correct trials, 70% incorrect trials, 70% correct trials. Seven seemed a valid number of preceding trials as this seems to be the plateau of repetition suppression (Jiang et al., 2000; Ulanovsky et al., 2004; Sayres and Grill-Spector, 2006), however, we have tested and confirmed this test with several amounts of preceding trials. Subsequently, we estimated the significance of the interaction effect [(30% incorrect trials-30% correct trials) versus (70% incorrect trials–70% correct trials)] via cluster statistics (Maris and Oostenveld, 2007; dependent samples T as dependent variable, using non-parametric cluster threshold at an alpha of 0.025, maxsum as dependent variable for the second level cluster analysis). Lastly, the behavioral analysis was repeated (at the final model of Experiment 1) including the factor SSVEP and the SSVEP interactions by calculating the *z*-score of the power for each individual.

#### Adaptation Effects – Between Subject

To investigate the SSVEP – temporal association relation over participants we correlated the accuracy difference between associated and non-associated sounds during the last block (temporal association effect) with the mean power over participants in the full session for all channels separately. Again, cluster statistics was used to analyze the significance of the correlation (using the correlation as the dependent variable, maxsum of the correlation for the second level analysis, alpha of 0.05). Influential cases were removed. These were defined as having a Cooks distance above 4/amount of participants (=0.1) for more than five channels. The removal of influential cases was repeated for each permutation in the cluster statistics.

## Results

### Experiment 1

One participant was removed for being an outlier (data was more than 1.5^*^inter-quartile range above the mean). The final model showed a significant Time^*^Association interaction (Figure 1C; *Z* = 2.071, *p* = 0.038, odds ratio = 1.07, [95%CI: 1.004–1.159]), a main effect of Sound Type (Z = −1.96, p = 0.050, odds ratio = 0.94, [95%CI: 0.883–0.999]), and a main effect of Categorization difficulty (*Z* = 2.347, *p* = 0.007, odds ratio = 1.06, [95%CI: 1.009–1.107]; total *r*^2^ of the fixed effects = 0.002). The random intercept showed a standard deviation of 0.129. To test for parsimony of the model, we compared the model to a model without any interaction. Indeed, the model with the interaction was better than the model without (χ^2^(1) = 4.294, *p* = 0.038). The interaction effect between sound type and association was not significant (*Z* = 0.807, *p* = 0.420, odds ratio = 1.06, [95%CI: 0.924–1.220]). None of the other effects were significant. We were mainly interested in the effect of Association over the course of the experiment. To test this, we estimated the main effect of Association at three different Time levels: at the beginning, in the middle, and at the end of the experiment. The main effect of Association in the full model is estimated at a level of zero for the time effect, therefore we re-ran the model centering Time at either the beginning, middle, or last trial (Figures 1D,E). At the beginning of the experiment no significant Association effect was found (*Z* = −0.731, *p* = 0.928, odds ratio = 0.95, [95%CI: 0.829–1.089]). However, in the middle of the experiment there was a trend for Association (*Z* = 2.113, *p* = 0.069, odds ratio = 1.08, [95%CI: 1.005–1.151]) and the end of the experiment participants were significantly better for sound presented at the 70% time point compared to the 30% time point (*Z* = 2.873, *p* = 0.008, odds ratio = 1.217, [95%CI: 1.064–1.391]). In sum, these analyses found no evidence that participants were generally better at one specific time point (no Sound Type^*^Association interaction), but showed that participants over the course of the experiment started responding more in line with the time-content associations.

### Experiment 2

#### Presentation Rate Determination

**FIGURE 2:**
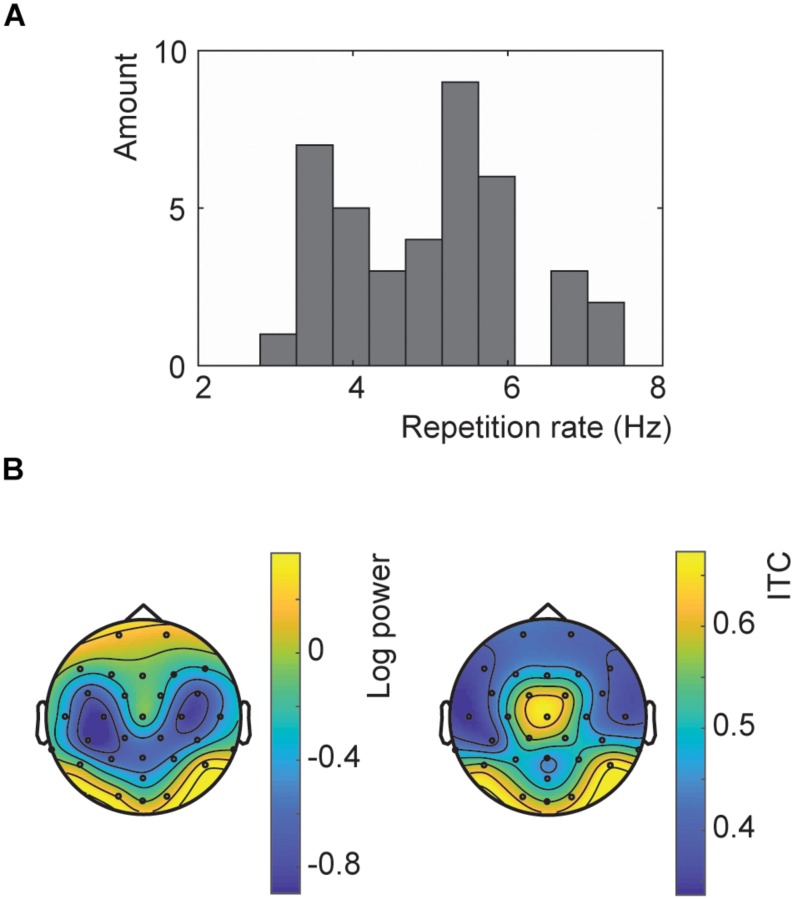
Results of the repetition rate optimization. **(A)** Histogram of all presentation rates used in Experiment 2. **(B)** Topographies of the SSVEP power (left) and ITC (right) for trials at the chosen presentation rates at neuronal frequencies matching these rates.

A wide variety of presentation rates were used in the main experiment ranging from 3 to 7.5 Hz (Figure 2A). The topography showed the strongest response at the occipital channels for both the SSVEP and ITC (Figure 2B). However, the ITC also showed a more central topography. However, this is likely influenced by the optimization of the ITC at exactly those channels.

#### Behavioral Analysis

**FIGURE 3:**
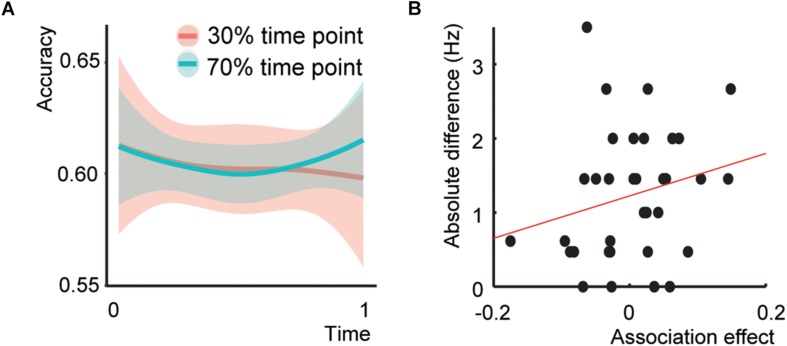
Behavioral results Experiment 2 and presentation rate – temporal association effect. **(A)** Behavioral results of Experiment 2. **(B)** Scatterplot between the association effect and the difference of the presentation rate with 4 Hz.

Five participants were classified as outliers. The model including the Time^*^Association interaction did not show a significant interaction (Figure 3A; *Z* = 0.407, *p* = 0.684, odds ratio = 1.014, [95%CI: 0.949–1.083]), a main effect of Association at the end of the experiment (*Z* = 0.397, *p* = 0.691, odds ratio = 1.028, [95%CI: 0.834–1.319]), or an interaction between Sound^*^Association (*Z* = −0.457, *p* = 0.647, odds ratio = 0.970, [95%CI: 0.849–1.107]). Only the effect of Sound was significant (*Z* = 2.75, *p* = 0.006, odds ratio = 1.089, [95%CI: 1.025–1.157], total *r*^2^ of the fixed effects = 0.011). Thus, Experiment 2 did not show the same initial Association effect as Experiment 1.

#### Presentation Rate

The addition of the main effect of Presentation rate and the Presentation Rate^*^Association interaction to the model did not result in any significant effect (interaction effect: *Z* = 0.815, *p* = 0.415, odds ratio = 1.028, [95%CI: 0.962-1.098]), nor a better fit (χ^2^(2) = 0.759, *p* = 0.684). The same held for the Presentation rate difference with 4 Hz. (Figure 3B; interaction effect: *Z* = 0.937, *p* = 0.349, odds ratio = 1.03, [95%CI: 0.966–1.103]) and fit χ^2^ (2) = 1.407, *p* = 0.495). This indicates that increasing or decreasing the exact presentation rate could not explain the absence of an association effect in Experiment 2.

#### Pure Temporal Predictions

Next, we investigated if the temporal distance of the time points to the expected time point within the rhythmic stream influenced the accuracy. If better performance was expected for in-phase stimuli a Phase Distance^*^Time Point interaction was expected. This interaction was not significant (*Z* = −1.639, *p* = 0.101, odds ratio = 0.72, [95%CI: 0.487–1.066]).

#### Adaptation – Within Subjects

**FIGURE 4:**
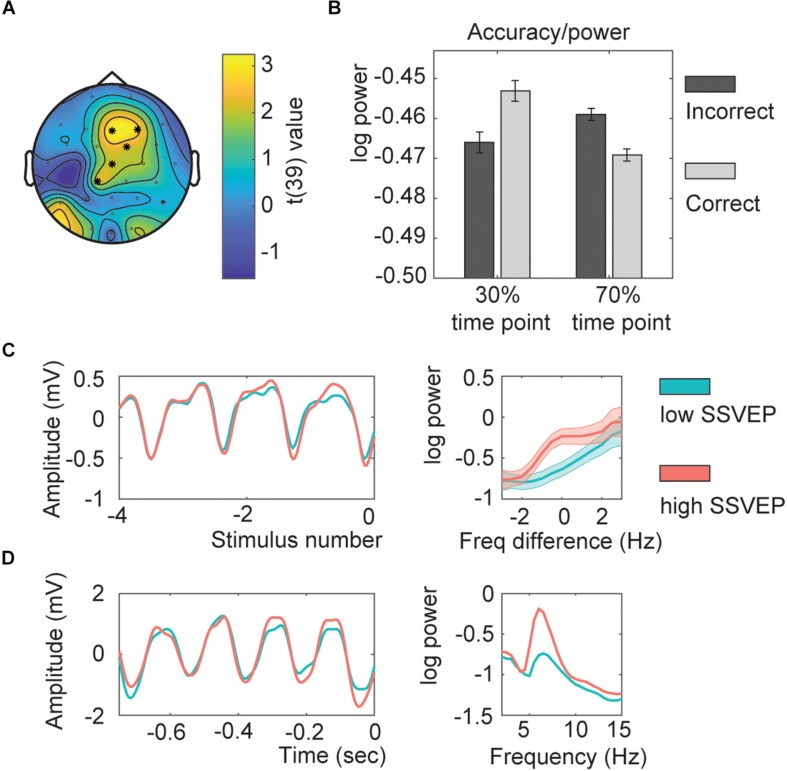
Within subject SSVEP power-association relation. **(A)** Topography of the *t*-values. Asterisks indicate the channels part of the significant cluster. **(B)** Interaction effect estimated for all channels part of the interaction. **(C)** Average time course for the SSVEP (aligned by stimulus number; left) and its corresponding power spectrum (0 represents the individual presentation rate; right). **(D)** Example of an SSVEP time course for one exemplar participant (left) with the corresponding power spectrum (right). All shaded lines and error bars represent the standard error of the mean.

The next analysis investigated if adaptation – as indexed by the strength of the neuronal responses to previous trials measured with the SSVEP – could explain the absence of a behavioral effect. The cluster analysis investigating the SSVEP power showed a significant effect for the interaction between accuracy and the time point (Figure 4A; clusterstatistics: 12.2265, *p* = 0.01). This cluster showed a fronto-central distribution. This result suggests that when the SSVEP were low, participants followed the temporal association; but when the SSVEP were high, participants did not follow the temporal association (Figure 4B). In a control analysis, we found that using between -13 up to -5 stimuli would have provided the same interaction (data not shown). This indicated that the SSVEP of the preceding trials had a significant impact on whether the participant would perform the current trial in line with the temporal association or not, and it was the fronto-parietal SSVEP that had the biggest influence. See Figures 4C,D for the SSVEP traces.

**FIGURE 5:**
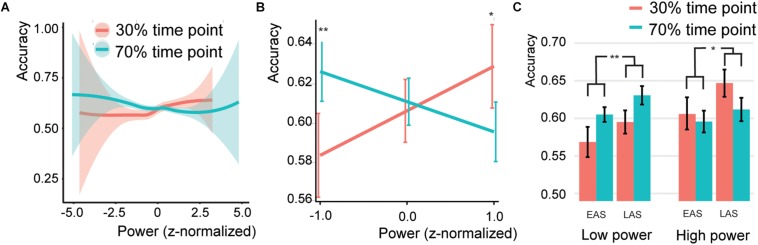
Association ^*^ SSVEP interaction **(A)** Behavior for different SSVEP power values. Shaded bars indicate the standard error of the mean. Note that the error bars at the extremes are high due to the low number of trials. **(B)** Predicted effects for the Association^*^SSVEP Power interaction. Error bars indicate the 95% confidence intervals. **(C)** Bar graphs for the association effect for both sound types (EAS = early association sounds, LAS = late association sounds), for the 50% low power and 50% high power trials. Error bars indicate the standard error of the mean. Asterisks and double asterisks indicate significance at the 0.05 and 0.005 level, respectively.

To investigate whether indeed participants followed the temporal association for low SSVEP trials as suggested with the previous analysis we repeated the behavioral analysis including the factor SSVEP in the model. If indeed for low amplitude SSVEP trials there was less adaptation, participants should follow the temporal association, being better for trials presented at their 70% time point (resulting in a SSVEP^*^Adaption interaction). The model including the factor SSVEP^*^Association was significantly better as a model with only main effects (χ^2^(2) = 19.66, *p* < 0.001). The effect of SSVEP^*^Association was significant (Figure 5A; *Z* = −4.428, *p* < 0.001, odds ratio = 0.853, [95%CI = 0.796–0.915]), as well as the main effect of Categorization difficulty (*Z* = 7.023, *p* < 0.001, odds ratio = 1.196, [95%CI = 1.138–1.125]), and Sound (*Z* = 2.880, *p* = 0.004, odds ratio = 1.099, [95%CI = 1.031–1.172], total *r*^2^ of the fixed effects = 0.012). None of the other interactions with SSVEP were significant.

The SSVEP^*^Association interaction was further investigated by extracting the association effect centered at a SSVEP *z*-score of −1, 0, or 1 (Figure 5B). This simple slope analysis investigated if at different levels of SSVEP amplitude the association effect was different (either following the association or not). It is common to estimate the effect of interest at values within the range of your own dataset, that is why *z*-scores of −1, 0, and 1 were chosen. For low SSVEP trials (estimated at a *z*-score centered at −1), participants were significantly better for associated sounds (*Z* = 3.509, *p* < 0.001, odds ratio = 1.195, [95%CI = 1.082–1.32]), for average SSVEP trials, no effect was found (*Z* = 0.543, *p* = 0.587, odds ratio = 1.019, [95%CI = 0.951–1.094]), and for high SSVEP (estimated at a *z*-score centered at 1) trials participants were significantly worse for associated sounds (*Z* = −2.753, *p* = 0.012, odds ratio = 0.870, [95%CI = 0.788–0.961]). The same was observed when doing two separate analyses for the 50% low and 50% high power trials. Higher accuracies for the 70% associated time point compared to the 30% associated time point for low-power SSVEP trials and vice versa for high-power SSVEP trials (Figure 5C; 50% low power: *Z* = 3.012, *p* = 0.005, odds ratio = 1.167, [95%CI = 1.055–1.289]; 50% high power: Z = −2.27, *p* = 0.052, odds ratio = 0.894, [95%CI = 0.810–0.988]).

#### Adaptation – Between Subjects

**FIGURE 6:**
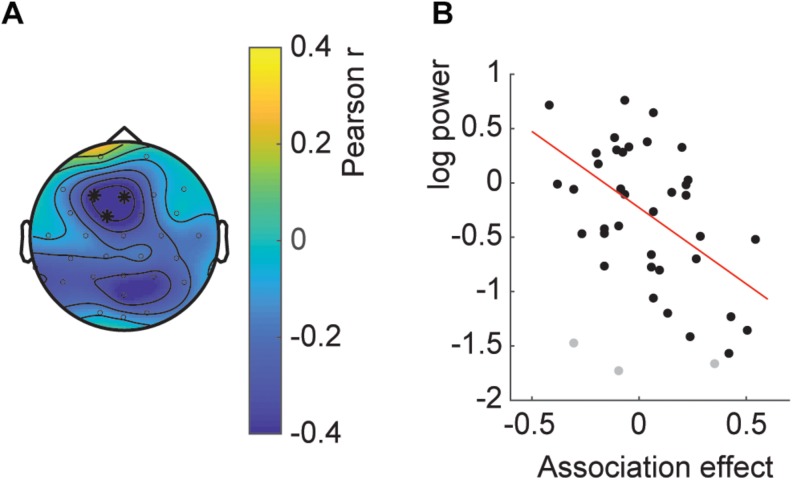
Between subject SSVEP power-association relation. **(A)** Topography of the correlation. Asterisks indicate the channels part of the significant cluster. **(B)** Scatterplot of the average SSVEP power per participant and the association effect. Gray dots indicate influential cases as described in the main text.

Last, we wanted to investigate whether the effect of decreased temporal associations with increased SSVEP also held over participants, that is, whether participants with high SSVEP had on average a lower temporal association. The correlation analysis showed a significant fronto-central cluster with a negative correlation between SSVEP and temporal association (Figure 6; clusterstatistics −9.996, *p* = 0.035; 3 influential cases detected). This correlation indicates that participants with a relatively high average SSVEP had a weaker or reversed association effect at the end of the experiment.

## Discussion

In the current study we investigated whether rhythmic temporal information influences behavioral responses toward the processing of any sensory input at expected time points, or whether instead parallel time-content predictions are created in which temporal information interacts with content information to optimally process a stimulus. To this end, we systematically presented different sound identities at different temporal delays after the offset of a visual rhythmic stimulus stream. We did this either with a fixed visual rhythmic rate of 4 Hz or with an individualized rhythmic rate based on the individual SSVEP response. In Experiment 1, we found that for the fixed visual rhythm, participants were indeed more accurate when a given sound content was presented at the associated time point. Initially, this was not replicated in the experiment with individualized rhythms. However, in this second experiment the association effect was modulated by SSVEP, that is, only trials preceded by trials with relatively low SSVEP showed this behavioral association effect. We interpret the current results as cautiously stating that temporal information can be used to enhance information processing related to a specific and expected content. Considering that the temporal-content association could only be found in Experiment 2 after a *post hoc* analysis we do believe that this requires future investigation.

We initially could not replicate the finding of Experiment 1 in Experiment 2. The main difference between these two experiments was the use of a fixed presentation rate in Experiment 1 versus an individualized rate in Experiment 2. We initially choose 4 Hz for Experiment 1 as previous studies have suggested that the brain is particularly sensitive to rates around 4-5 Hz (Poeppel, 2003; Ghitza, 2013; Santoro et al., 2017). To investigate if the deviation from 4 Hz could indeed explain the absence of an effect we performed a *post hoc* analysis in which we added the factor presentation rate difference to 4 Hz to the model. This factor did not show a significant effect and thus provided no evidence that 4 Hz is a superior rhythmic frequency.

A second reason why individualized frequencies might remove the association effect is because increasing neuronal sensitivity to the stimulation could lead to adaptation after repeated presentation. Specifically, it has been shown that when a stimulus is presented repeatedly, it’s corresponding neuronal response decreases (Nelson, 1991; Sobotka and Ringo, 1994; Larsson and Smith, 2011). These effects can last from seconds (Ulanovsky et al., 2004) up to several minutes (Schweinberger et al., 2008). The repetition suppression is typically stronger when neuronal populations are more sensitive to the incoming input (Li et al., 1993; Sobotka and Ringo, 1994). Repetition suppression is sensitive to both the time of presentation (Tse et al., 2004; Van Wassenhove et al., 2008) as well as to the exact features that are repeated (Doniger et al., 2001; Schwartz et al., 2007). After adapting to a stimulus, subsequent behavioral responses to ambiguous stimuli show increased choices inconsistent with the adaptor (Kanai and Verstraten, 2005; Daelli et al., 2010). To test for this adaptation effect we included the amplitude of the SSVEP in the previous trials as a *post hoc* analysis in the model. This SSVEP amplitude influenced whether participants responded in accordance or not with the time-content association. Specifically, participants followed the time-content association for low SSVEP trials, but responded inconsistent with the association for high SSVEP trials. Note that this effect cannot be explained by modulation of attention to the auditory or visual stimulation depending on the SSVEP amplitude as the overall accuracy of the participant did not differ between low versus high SSVEP trials. However, we have to be cautious with the interpretation as the current analysis was part a *post hoc* descriptive analysis and not intended to test for adaptation effects, limiting the weight of the results. Considering the vast amount of research in the field of entrainment and temporal/content prediction (Haegens and Golumbic, 2017), we did, however, feel that the current results would significantly contribute to the development of optimized designs. Specifically, adaptation effects need to be taken into account for any study design, as they might lower the effect sizes, or even abolish or reverse the expected effect.

In our study the probability of sound type and time point was identical, with only the combinatory sound-time constellation having an imbalanced probability. Therefore, the adaptation did not only integrate the specific sound type, but also the time of the stimulus presentation, creating a sound-time specific adaptation. Adaptation of time-of-presentation has been reported in oddball paradigms where repeating the same delays reduces the neuronal response (Tse et al., 2004; Van Wassenhove et al., 2008). A previous study investigating spectral and temporal oddballs separately suggested a different lateralization for these two oddballs (Zaehle et al., 2009), making any possible interaction relatively complex. But for other sensory features (audio-visual stimuli) the oddball response has been shown to interact (Ullsperger et al., 2006). Whether this interaction would also occur for acoustic and temporal features is not evident and the currently described adaptation to a specific sound-time combination is an open field for future research.

Given the significant effect of association at the end of Experiment 1 and the significant effect of association after controlling for adaptation in Experiment 2 we cautiously interpret these effect as showing that participants use temporal delays to guide sound categorization when sound identity is systematically mapped to these delays. This signifies that it is possible to keep track of multiple time-content associations, adding new findings to the previous literature suggesting that time and feature predictions interact (Zaehle et al., 2009; Rimmele et al., 2011; Ten Oever et al., 2013; Morillon et al., 2016). Most of these previous studies have focused on the presence or absence of predictions instead of keeping track of multiple predictions, rendering any inference about parallel time-content predictions problematic. While our study shows behavioral effects of time-content predictions, it cannot be excluded that participants use time itself as a trigger to activate specific content, thereby not having parallel, but sequential representations. We believe this explanation unlikely as previous studies have shown that information needed at a future time point is available earlier (Rose et al., 2016; Wolff et al., 2017). What can be concluded is that time is dynamically used to infer content based on statistical regularities. However, future studies need to dissociate whether association information is used to bias perception, to bias decision making, or to bias both.

Finding a time-content association within the temporal range in this study would indicate that temporal associations for different content can be made even within one cycle of a rhythmic input stream. Such a finding provides important constraints to neuronal models for rhythmic processing. Previously, (cross-modal) neuronal populations have been found to align their oscillatory phase to rhythmic input structure (Lakatos et al., 2005; Besle et al., 2011). This alignment has been thought of as a temporal prediction mechanism which ensures that information occurring within the rhythmic structure falls on the most excitable phase of the oscillation, thereby promoting its neuronal processing (Schroeder and Lakatos, 2009). If this type of entrainment occurs during the current paradigm, it is not possible that information always falls on the most excitable phase (as the phase distance between the time points were maximized) unless neighboring neuronal populations coding for different sounds are at their most excitable phase at different time points. Hence, alignment for a rhythmic temporal structure has to occur at different phases for different neuronal populations. Indeed, it has been shown that entrainment to rhythmic input only occurs in sensory populations specifically involved in the upcoming behavioral task (O’Connell et al., 2011, 2014; Lakatos et al., 2013). However, so far no study has shown that entrainment occurs at different phases for different neuronal populations dependent on temporal occurrence of the task-relevant input. Alternatively, there is not one optimal, most sensitive phase, but phase is used as a cue for specific content (Jensen et al., 2014; Watrous et al., 2015; Bahramisharif et al., 2018) as would be predicted by models suggesting that high frequency oscillations nested in low frequencies represent information content (Jensen et al., 2012; Lisman and Jensen, 2013). Indeed, we have previously suggested that temporal information might be encoded by the phase of ongoing oscillations (Ten Oever and Sack, 2015). Others have also proposed that time is inherently part of the representational space of a stored object (Gallistel and Balsam, 2014; Molet and Miller, 2014). However, future research is needed to verify this hypothesis.

In the current study we used a continuous staircase procedure to maintain task difficulty. The neuronal populations encoding for the two types of sounds/content are consequently very similar and widely overlapping. Alternatively, we could have added noise to the auditory stimuli to make the task more difficult. In this way, the neuronal populations representing more extreme content would be less overlapping. It is unclear which strategy would have led to the strongest time-content associations. One could argue that having a clearer differentiation between the neuronal populations would improve the associations between temporal information and content. However, one could also argue that the more similar the neuronal populations that represent two different contents, the more important it is for the system coding these representations to use extra information in the environment to separate the representations. To dissociate these two options, future research is needed.

## Conclusion

In everyday live we are bombarded with an abundance of sensory information. In order to make sense of all this information it is beneficial to extract as much statistical regularities as possible (Summerfield and Egner, 2009; Schroeder et al., 2010). In this viewpoint temporal information should not merely be a cue for when (any) input can be expected, but should trigger a cascade of parallel predictions relating to other features of the predicted content. Here we investigated how time can be used as a cue for content. How this time-feature information is coded in the brain is still an open question (but see Ten Oever and Sack, 2015). Furthermore, we show that this type of learning might be prone to adaptation (Ulanovsky et al., 2004), being modulated by the strength of the neuronal response of the preceding trials. This latter result shows that the behavioral effects found for rhythmic input are modulated by adaptation mechanisms; a finding which could potentially explain several behavioral null-results known in the field. Future research should further investigate time-content associations as well as the influence of adaptation on this effect.

## Data Availability

All data presented in this paper are available online (https://hdl.handle.net/10411/GSGDSV). Access to the datasets is available on request to the corresponding author.

## Ethics Statement

This study was carried out in accordance with the recommendations of the local ethical committee at Faculty of Psychology and Neuroscience Maastricht University with written informed consent from all subjects. All subjects gave written informed consent in accordance with the Declaration of Helsinki. The protocol was approved by the local ethical committee at Faculty of Psychology and Neuroscience Maastricht University.

## Author Contributions

StO designed and analyzed the study and wrote the manuscript. AS contributed to the conception and design, and wrote the manuscript.

## Conflict of Interest Statement

The authors declare that the research was conducted in the absence of any commercial or financial relationships that could be construed as a potential conflict of interest.
